# Purulent MRSA Pericarditis Secondary to Chronic Diabetic Foot Osteomyelitis: A Case Report

**DOI:** 10.1155/crdi/7744624

**Published:** 2026-10-16

**Authors:** Maya Itani, Gustavo Rivera-Alvarez

**Affiliations:** ^1^ Department of Emergency Medicine HCA Florida Lawnwood Fort Pierce Florida USA; ^2^ Department of Critical Care Medicine HCA Florida Lawnwood Fort Pierce Florida USA

**Keywords:** cardiac tamponade, case report, methicillin-resistant *Staphylococcus aureus*, osteomyelitis, purulent

## Abstract

Purulent pericardial effusion is a rare but life‐threatening manifestation of bacterial infection, most commonly caused by Staphylococcus aureus bacteremia. We report the case of an 86‐year‐old female with chronic diabetic foot osteomyelitis complicated by persistent methicillin‐resistant *Staphylococcus aureus* (MRSA) bacteremia. The patient subsequently developed purulent pericarditis, bilateral exudative pleural effusions, septic shock, and acute renal failure requiring continuous renal replacement therapy (CRRT) after intolerance to intermittent hemodialysis due to refractory hypotension. Despite broad‐spectrum antimicrobial therapy and drainage of infected compartments, the patient developed refractory multiorgan failure and expired on hospital Day 26. This case highlights the high mortality associated with purulent pericarditis in the setting of persistent bacteremia and delayed source control.

## 1. Introduction

The pericardium is a fibroserous sac enclosing the heart and proximal great vessels. Under normal physiologic conditions, the pericardial space contains up to 50 mL of serous fluid that functions as a lubricant, reducing friction during cardiac motion. Pericardial effusion refers to an abnormal accumulation of fluid within this space and may arise from various etiologies [1]. Clinical severity ranges from asymptomatic incidental findings to life‐threatening cardiac tamponade [2].

Infectious causes of pericardial effusions are commonly caused by tuberculosis in developing countries; however, in developed countries, this is less common, with other microorganisms presenting as purulent pericarditis [3]. Bacterial causes stem from direct infection secondary to trauma, thoracic surgery, catheter drainage, or hematogenous dissemination. *Staphylococcus aureus* and *Streptococcus species* remain among the most frequently identified pathogens in purulent pericarditis [4].

Purulent pericarditis is the most severe form of bacterial pericardial infection and is characterized by the accumulation of grossly purulent fluid within the pericardial space. It is a rapidly progressive condition associated with significant morbidity and mortality despite advances in antimicrobial therapy and critical care management. Clinical presentation is often nonspecific, with fever and systemic signs of infection more commonly observed than classic pleuritic chest pain. Prompt recognition and urgent drainage, in addition to intravenous antimicrobial therapy, are essential to improving outcomes [4]. Delayed diagnosis may result in cardiac tamponade, septic shock, multiorgan failure, and death.

We present a case of persistent methicillin‐resistant *Staphylococcus aureus* (MRSA) bacteremia secondary to chronic osteomyelitis complicated by purulent pericarditis, refractory septic shock, and renal failure requiring CRRT.

## 2. Case Presentation

An 86‐year‐old female with a history of Type 2 diabetes mellitus complicated by chronic left foot osteomyelitis, hypertension, and hyperlipidemia initially presented to an outside hospital after home health nursing noted increased drainage from a chronic plantar wound during dressing changes. She was admitted for chronic osteomyelitis with superimposed systemic infection. Table 1 summarizes the timeline of her hospital course and major therapeutic interventions.

**TABLE 1:** Clinical timeline of the hospital course and major therapeutic interventions.

Hospital day	Clinical event	Antimicrobial & interventions	Diagnostic data and notes
HD 1–HD 7	Admitted for chronic foot ulcer and osteomyelitis.	• IV Vancomycin + cefepime started (HD 2). • Cefepime switched to meropenem (h/o ESBL). • Surgical consult obtained and deferred	• HD 1: Blood cultures + for MRSA (x 2). • HD 4 and HD 6: Persistent MRSA bacteremia. • HD 6: TTE negative for vegetations.
HD 10	Worsening acute kidney injury.	• Transitioned from vancomycin to Daptomycin.	• Persistent MRSA bacteremia on serial cultures.
HD 14–HD 18	Respiratory failure; ICU transfer.	• HD 16: Initiated midodrine. • HD 18: Added linezolid to daptomycin.	• Imaging: Pneumonia and bilateral pleural effusions.
HD 19–HD 21	Hemodialysis failure; transfer to tertiary facility.	• HD 19: Intermittent HD recommended. • HD 21: Failed trial HD 2/2 hypotension; initiated CRRT.	• Progressive multiorgan dysfunction.
HD 22–HD 25	Purulent pericardial tamponade.	• HD 24: Bedside pericardiocentesis (450 mL purulent fluid drained). • HD 25: Escalated to ceftaroline + daptomycin. • HD 25: Placement of bilateral pigtail catheters	• HD22: Blood Cx drawn • HD 23: TTE confirms tamponade. • HD 23: Last dose of linezolid. • HD 24: Repeat TTE negative for vegetations (TEE deferred 2/2 instability). • HD 25: Susceptibilities result • HD 25: Pleural fluid analysis consistent with exudative effusions
HD 26	Clinical expiration.	• Final antibiotic doses given (daptomycin, ceftaroline).	• Refractory septic shock; delayed source control likely contributed to persistent bacteremia and progressive multiorgan dysfunction.

On presentation (Hospital Day 1), she was hemodynamically stable but demonstrated significant leukocytosis and elevated inflammatory markers. She was initiated on empiric vancomycin and cefepime; however, she was switched for meropenem due to a history of ESBL infection. Initial blood cultures subsequently grew MRSA, and wound cultures confirmed MRSA from the chronic foot ulcer, consistent with a persistent infectious source with hematogenous dissemination. Surgical consult was obtained for debridement of the foot wound which was deferred due to patient instability.

Despite targeted antimicrobial therapy, repeat blood cultures remained persistently positive for MRSA. During her hospital course at the transferring facility, bilateral pleural effusions were identified and managed with thoracentesis; fluid analysis at this time was negative for active infection. Her hospital course was complicated by worsening sepsis with acute kidney injury, declining urine output, and progressive multiorgan dysfunction. At this time (Hospital Day 10), vancomycin was replaced with daptomycin due to worsening renal function.

She subsequently developed septic shock requiring initiation of vasopressor support. In the setting of worsening renal failure and hemodynamic instability, intermittent hemodialysis was attempted; however, it was not tolerated due to refractory hypotension. Given progressive renal failure and inability to maintain adequate hemodynamic stability, she was transferred to our facility for initiation of continuous renal replacement therapy (CRRT). On Hospital Day 18, linezolid was added alongside daptomycin due to the persistent bacteremia and progressive clinical decline.

Upon arrival to our institution on Hospital Day 21, the patient remained critically ill with ongoing vasopressor requirements. Repeat blood cultures drawn on Hospital Day 22 confirmed persistent bacteremia. Leukocytosis persisted despite broad‐spectrum antimicrobial therapy. CRRT was initiated; however, the patient continued to demonstrate escalating vasopressor requirements and worsening multisystem organ dysfunction. Persistent bacteremia raised concern for an uncontrolled source of infection despite antimicrobial therapy.

Transthoracic echocardiography was repeated at our facility and demonstrated a large pericardial effusion with preserved left ventricular systolic function (Figure 1) with no clear vegetations. Transesophageal echocardiography was deferred due to profound hemodynamic instability.

**FIGURE 1:**
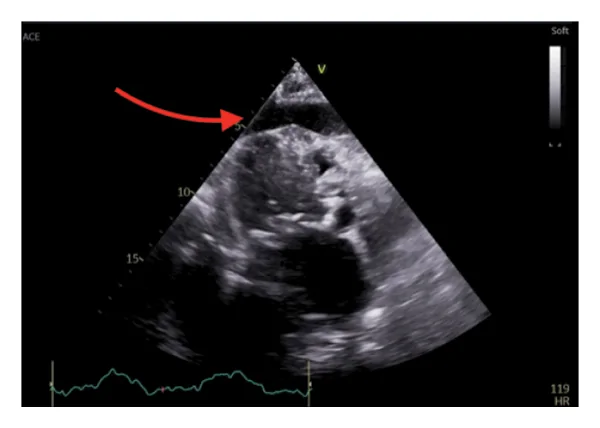
Transthoracic echocardiogram demonstrating significant pericardial effusion (red arrow).

Cross‐sectional imaging with CT chest further confirmed a significant pericardial effusion in addition to bilateral pleural effusions, suggesting disseminated infection involving multiple serosal compartments (Figure 2).

**FIGURE 2:**
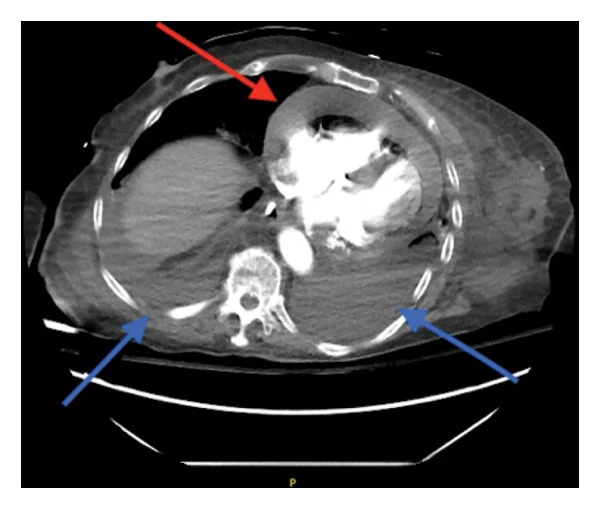
CT chest demonstrating significant pericardial effusion with ventricular collapse (red arrow) and bilateral pleural effusions (blue arrows).

Given hemodynamic instability and echocardiographic findings consistent with cardiac tamponade, ultrasound‐guided pericardiocentesis was performed at the bedside for therapeutic relief, yielding approximately 450 mL of grossly purulent fluid (Figure 3).

**FIGURE 3:**
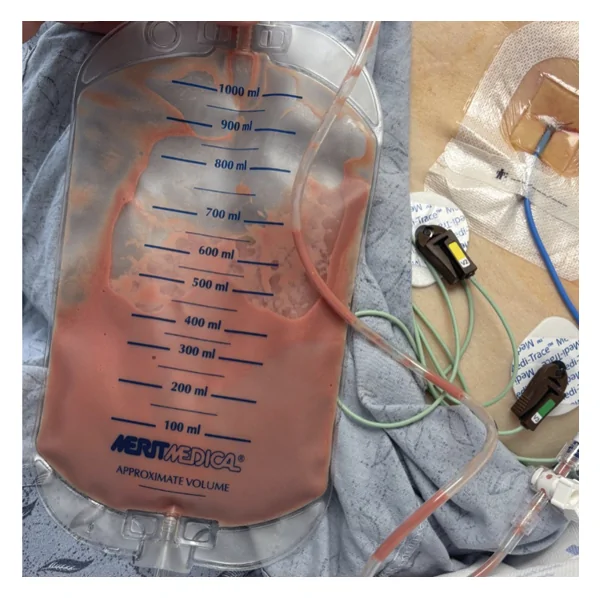
Pericardial drain placement with 450 mL of purulent pericardial fluid drained on initial insertion.

Pericardial fluid cultures grew MRSA, confirming purulent pericarditis in the setting of persistent MRSA bacteremia. A pericardial window was considered; however, it was decided against, given the patient’s hemodynamic instability.

On Hospital Day 25, bilateral pleural effusions, as noted on the CT chest (Figure 2), were managed with the placement of bilateral pigtail catheters. Pleural fluid analysis was consistent with exudative effusions.

Cultures drawn on Hospital Day 22 had resulted in susceptibility on Hospital Day 25, demonstrating MRSA susceptibility to vancomycin (MIC 1 µg/mL), daptomycin (MIC 0.25 µg/mL), and ceftaroline (MIC 1 µg/mL). Antimicrobial coverage was escalated to include ceftaroline alongside daptomycin, in an effort to achieve clearance of the persistent bacteremia; however, clearance was not achieved. Despite multiagent antimicrobial therapy, vasopressor support, CRRT, and multiple attempts at source control, the patient developed refractory septic shock with progressive multiorgan failure and ultimately expired on Hospital Day 26.

## 3. Discussion

This case highlights a rare but severe complication of persistent MRSA bacteremia with a likely source of osteomyelitis from a diabetic foot wound. Although uncommon, bacterial pericarditis has been reported and is most often caused by Gram‐positive cocci, with *Staphylococcus aureus* accounting for over 30% of cases [4].

A significant management challenge in this case was navigating severe dialysis‐dependent acute kidney injury requiring CRRT during refractory septic shock. Given echocardiographic features of cardiac tamponade, ultrasound‐guided pericardiocentesis was performed for therapeutic relief, with transient hemodynamic improvement. However, the pericardial drainage was positive for MRSA, consistent with her blood cultures and foot wound, despite ongoing antimicrobial therapy.

Literature review demonstrates several reported cases of MRSA purulent pericarditis, typically arising in patients with underlying immunosuppression, cardiac devices, central venous catheters, or underlying structural heart disease [5–8]. Our patient had a medical history pertinent to diabetes, which is a known predisposing risk factor for invasive bacterial infection and purulent pericarditis. She also had a known source of infection on initial presentation.

Early diagnosis and treatment of MRSA purulent pericarditis is crucial, as mortality in untreated patients approaches 85%. Treatment with vancomycin, alongside drainage, is mandatory in these cases [4]. In our case, combination therapy with daptomycin and linezolid was initiated due to persistent MRSA bacteremia and pulmonary involvement, later escalated to ceftaroline. Linezolid provided nonrenal clearance kinetics during CRRT and superior tissue penetration into pleural spaces, while ceftaroline provided bactericidal clearance for refractory MRSA [9].

Despite appropriate antimicrobial therapy and eventual drainage of infected spaces, delayed source control in persistent bacteremia likely contributed to ongoing multiorgan failure [5, 6]. Purulent pericarditis remains a condition with high mortality, particularly when associated with septic shock and renal failure.

## 4. Conclusion

Purulent pericardial effusion is a rare but life‐threatening complication of disseminated bacterial infection, most commonly seen with *Staphylococcus aureus* bacteremia. This case highlights the importance of maintaining a high index of suspicion in patients with persistent bacteremia and multiorgan dysfunction, particularly when clinical deterioration persists despite adequate antimicrobial therapy. Early recognition and adequate source control remain critical; however, mortality remains high in patients presenting with septic shock and end‐organ failure.

## Author Contributions

Conceptualization: Maya Itani and Gustavo Rivera‐Alvarez. Data curation: Maya Itani. Writing–original draft: Maya Itani. Writing–review and editing: Maya Itani and Gustavo Rivera‐Alvarez.

## Funding

No funding was received for this manuscript.

## Disclosure

This research was supported (in whole or in part) by HCA Healthcare and/or an HCA Healthcare‐affiliated entity. The views expressed in this publication represent those of the authors and do not necessarily represent the official views of HCA Healthcare or any of its affiliated entities.

## Ethics Statement

Institutional Review Board (IRB) approval was waived by our institution, as case reports involving single, fully anonymized patient presentations do not meet the definition of human subjects research.

## Consent

The patient described in this report is deceased. All reasonable efforts to contact the next of kin to obtain written informed consent for publication were unsuccessful. This case has been thoroughly anonymized to ensure that neither the individual nor any acquaintances can identify them. All clinical data presented are limited to those strictly necessary for the scientific and educational purposes of this report.

## Conflicts of Interest

The authors declare no conflicts of interest.

## Supporting Information

Additional supporting information can be found online in the Supporting Information section.

## Supporting information


**Supporting Information** Care Checklist Itani Updated.

## Data Availability

Data sharing is not applicable to this article as no datasets were generated or analyzed during the current case report. All relevant clinical information is included within the manuscript.
